# Association between Genetic Polymorphisms and Bleeding in Patients on Direct Oral Anticoagulants

**DOI:** 10.3390/pharmaceutics14091889

**Published:** 2022-09-07

**Authors:** Ha-Young Yoon, Tae-Jin Song, Jeong Yee, Junbeom Park, Hye-Sun Gwak

**Affiliations:** 1 College of Pharmacy and Graduate School of Pharmaceutical Sciences, Ewha Womans University, 52 Ewhayeodae-gil, Seodaemun-gu, Seoul 03760, Korea; 2 Department of Neurology, Ewha Womans University Seoul Hospital, Ewha Womans University College of Medicine, Seoul 07804, Korea; 3 Division of Cardiology, Department of Internal Medicine, Ewha Womans University Mokdong Hospital, Ewha Womans University College of Medicine, Seoul 07985, Korea

**Keywords:** DOAC, ABCB1, CYP3A5, APOB, APOE, risk scoring system, bleeding, genetic polymorphisms

## Abstract

**Objectives**: The purpose of our study is to investigate the effects of apolipoprotein B (*APOB*) and *APOE* gene polymorphisms on bleeding complications in patients receiving direct oral anticoagulants (DOACs). **Methods**: A total of 16 single nucleotide polymorphisms (SNPs) in 468 patients were genotyped. Six SNPs of *ABCB1* (rs3842, rs1045642, rs2032582, rs1128503, rs3213619, and rs3747802), one SNP of *CYP3A5* (rs776746), seven SNPs of *APOB* (rs1042034, rs2163204, rs693, rs679899, rs13306194, rs13306198, and rs1367117), and two SNPs of *APOE* (rs429358 and rs7412) were analyzed by a TaqMan genotyping assay. Multivariable logistic regression analysis with selected variables was performed for the construction of a risk scoring system. Two risk scoring systems were compared (demographic factors only vs. demographic factors and genetic factors). **Results**: In the multivariable analyses, two models were constructed; only demographic factors were included in Model I and both demographic factors and genetic factors in Model II. Rivaroxaban and anemia showed significant association with bleeding in both models. Additionally, *ABCB1* rs3842 variant homozygote carriers (CC) and *APOB* rs13306198 variant allele carriers (AG, AA) had a higher risk of bleeding risk compared with that of wild-type allele carriers (TT, TC) and wild-type homozygote carriers (GG), respectively. Whereas the area under the receiver operating characteristic curve (AUROC) value using demographic factors only was 0.65 (95% confidence interval (CI): 0.56–0.74), the AUROC increased to 0.72 by adding genetic factors (95% CI: 0.65–0.80). The predicted bleeding risks of bleeding in patients with 0, 1, 2, 3, 4, 5, 6, 7 and 8 points from the logistic regression curve were 0.8%, 2.0%, 5.4%, 5.2%, 12.5%, 26.9%, 47.0%, 64.3% and 82.3%, respectively. **Conclusions**: The study results can be used for enhancing individualized treatment strategies in patients taking DOACs, helping clinicians predict the bleeding risk.

## 1. Introduction

Direct oral anticoagulants (DOACs) are important for the treatment of non-valvular atrial fibrillation (NVAF) and venous thromboembolism (VTE) [1]. A high inter-individual variability in drug blood levels has been observed with all DOACs, which may result in bleeding or thrombotic events. Several non-genetic factors accounting for the high variability between individuals in DOAC treatments have been identified (e.g., age, weight and renal dysfunction) [2,3]. However, differences still remain largely unexplained and genetic factors may play an important role.

All DOACs are substrates of the efflux transporter permeability-glycoprotein (P-gp, also referred to as ABCB1) [4]. Moreover, some DOACs such as apixaban, edoxaban and rivaroxaban are known to be metabolized by cytochrome P450 (CYP) 3A5 [5,6]. Several genetic polymorphisms have been reported to influence ABCB1 and CYP3A5 activity and/or expression [7,8,9,10].

Various studies have shown that apolipoproteins are involved in platelet function, the abnormal function of which leads to arterial thrombosis and bleeding complication [11,12]. In particular, apolipoprotein B (APOB)-100 component of low-density lipoprotein binds to a receptor on the platelet membrane and sensitizes platelets. Apolipoprotein E (APOE)-containing lipoproteins, such as high-density lipoproteins, mediate the inhibition of platelet function [13]. Recent publications also showed that apolipoproteins were related to hemorrhage [14,15]. However, no studies have been conducted on the effect of these gene polymorphisms on bleeding complications during DOAC treatment.

As bleeding is one of the most severe complications of DOACs, bleeding risk should be assessed during treatment [16]. However, existing scores or calculators for predicting bleeding risks have been primarily validated or evaluated using VKAs and parenteral anticoagulants [17]. Because DOACs have different metabolic pathways from VKAs, it is necessary to consider DOAC-related genetic polymorphisms rather than VKA-related genetic polymorphisms (e.g., CYP2C9*3) [18]. Therefore, bleeding risk scores for DOACs with high accuracy are necessary. The primary objective of this study was to investigate the association between genetic polymorphisms including *ABCB1*, *CYP3A5*, *APOB*, and *APOE*. The secondary objective was to construct an optimal risk scoring system for bleeding complications in patients receiving DOACs.

## 2. Methods

### 2.1. Study Patients and Data Collection

This study was a retrospective analysis of prospectively collected samples from June 2018 to December 2021. It was conducted at Ewha Womans University Mokdong Hospital and Ewha Womans University Seoul Hospital. This study was approved by the Institutional Review Board (IRB) of each hospital in accordance with the 1975 Helsinki Declaration and its later amendments (IRB number: 2018-04-006 and 2019-05-038, respectively). Written informed consent was obtained from all participants before enrollment.

The subjects of this study were those aged ≥ 20 years old and who received DOACs (apixaban, edoxaban, rivaroxaban or dabigatran). Patients who had thromboembolic or infarction-related events during the follow-up period experienced bleeding that was minor or unverified by health professionals while on treatment, and those who experienced bleeding after 1 year of DOAC therapy were excluded. In the case of the control group, patients treated with DOACs for less than 3 months were also excluded. The primary endpoint was major bleeding and clinically relevant non-major bleeding (CRNMB), according to the criteria of the International Society on Thrombosis and Haemostasis (ISTH) [19,20].

Data were obtained from electronic medical records. Patient demographics, including sex, age, body mass index (BMI), creatinine clearance (CrCl) estimated by the Modification of Diet in Renal Disease (MDRD) formula, aspartate transaminase (AST), alanine transferase (ALT), type and prescription dose of the DOAC used, concurrent medication, any history of myocardial infarction, stroke, transient ischemic attack, thromboembolism, or bleeding, comorbidities, smoking status and alcohol status were collected. The CHA_2_DS_2_-VASc (congestive heart failure, hypertension, age ≥ 75 years, diabetes mellitus, stroke, vascular disease, age 65–74 years and sex category; range 0–9) score, which is used for stroke risk assessment, and modified HAS-BLED (hypertension, abnormal renal or liver function, stroke, bleeding history or predisposition, elderly (age ≥ 65 years) and concomitant drug and alcohol use; range 0–8) score, which is a specific risk score designed for bleeding risk assessment, were calculated from its component variables [21,22].

### 2.2. Selection of Single Nucleotide Polymorphisms (SNPs) and Genotyping

To select SNPs of DOAC-associated genes, Haploreg 4.1 for minor allele frequencies, functionality, and linkage disequilibrium (LD) patterns of SNPs in Asian populations was used [23]. In this study, one missense SNP (rs2032582), two synonymous SNPs (rs1045642 and rs1128503), one 3ʹ-untranslated region (UTR) SNP (rs3842) and two 5ʹ-UTR SNPs (rs3213619 and rs3747802) were selected from *ABCB1*, and one splice donor SNP (rs776746) was selected from *CYP3A5*. Six missense SNPs (rs1042034, rs2163204, rs679899, rs13306194, rs13306198 and rs1367117) and one synonymous SNP (rs693) were selected from *APOB* and two missense SNPs (rs429358 and rs7412) were selected from *APOE*. Finally, a total of 16 SNPs were investigated.

The genomic deoxyribonucleic acid (DNA) of the patients was extracted from the blood or saliva. Genomic DNA was extracted from EDTA blood samples using the QIAamp DNA Blood Mini Kit (QIAGEN GmbH, Hilden, Germany). Otherwise, saliva samples were collected from OraGene-600 kits (DNA Genotek, Ottawa, ON, Canada) and subjected to genomic DNA extraction with PrepIT reagents (DNA Genotek, Ottawa, ON, Canada). The genotypes of 16 SNPs were analyzed by TaqMan assay.

### 2.3. Statistical Analysis

Unpaired *t*-tests were used to compare continuous variables between patients who experienced bleeding and those who did not. Chi-squared test and Fisher’s exact test were used to analyze categorical variables. A multivariable logistic regression model was used to identify independent risk factors for bleeding after adjusting for variables with *p* < 0.05 in univariate analysis. Variables were entered by stepwise selection when *p* was lower than 0.05 and were removed when *p* was higher than 0.1. The unadjusted odds ratio (OR) and adjusted OR with the 95% confidence interval (CI) were calculated from univariate and multivariable analyses, respectively. To test the fit of the prediction model, the Hosmer–Lemeshow goodness-of-fit test was performed. We constructed two models using demographic factors only and both demographic and genetic factors. The discrimination of the model was further evaluated by calculating the area under the receiver operating characteristic curve (AUROC).

Using the model with better accuracy, the risk scoring system was constructed by dividing the adjusted OR by the smallest adjusted OR among the variables. Then, quotients were rounded to the nearest integer to develop a risk scoring system for predicting the bleeding risk. The bleeding risk predicted by logistic regression analysis was compared with the observed risk. All analyses were based on two-tail statistics and were performed using the Statistical Package for Social Sciences version 20.0 (IBM Corp., Armonk, NY, USA). *p* < 0.05 was considered statistically significant.

## 3. Results

**Figure 1:**
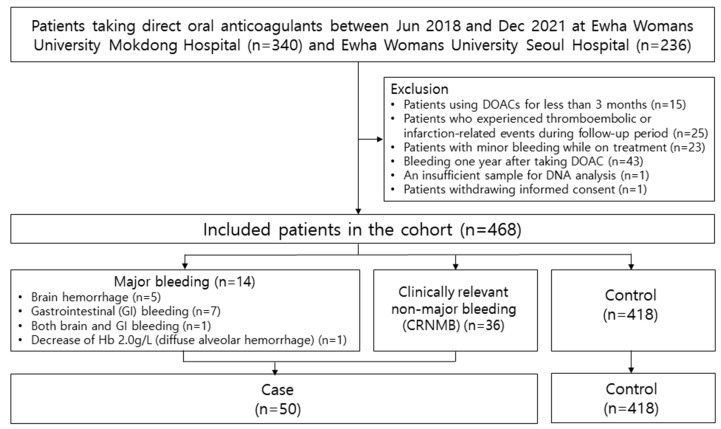
Patient flowchart.

A total of 576 patients were selected for the study (Figure 1). Overall, 15 patients who had been treated with DOACs for <3 months, 25 patients who had a history of thromboembolic or infarction-related events, 23 patients who had reported minor bleeding that could not be verified by health professionals, 43 patients who had any bleeding at least 1 year after DOAC therapy, 1 patient with a sample that was insufficient for DNA analysis and 1 patient who withdrew informed consent were excluded. Finally, 468 patients were included in the analysis. A total of 50 patients (10.7%) experienced bleeding complications, of whom 14 patients had major bleeding and 36 patients had CRNMB. The time (mean ± standard deviation) to bleeding event was 110.04 ± 11.45 days.

Table 1 shows the demographic and clinical characteristics of the study population. The mean age of the included patients was 69.2 years, and 293 patients (62.5%) were male. Apixaban was the most prescribed DOAC, followed by edoxaban. Approximately, a third of the patients received an underdose of DOACs. Among the co-medications, the most common drug class was beta-blockers, followed by statins. Approximately 98% of the patients had atrial fibrillation, and approximately 70% of the patients had hypertension. Type of DOACs, dose of DOACs and anemia were significant factors for bleeding complications.

In grouped genotype analysis, variant homozygote carriers (CC) of *ABCB1* rs3842 had a higher bleeding risk compared with that of wild-type allele carriers (20.4% vs. 9.5%, *p* = 0.02) (Table 2). Wild-type homozygote carriers (GG) of *APOB* rs693 had a higher bleeding risk compared with that of variant allele carriers (11.7% vs. 2.0%, *p* = 0.04) as shown in Table 3. In addition, variant allele carriers (AG, AA) of *APOB* rs13306198 had a greater risk of bleeding compared with that of wild-type homozygote carriers (GG) (26.0% vs. 8.9%, *p* < 0.01).

Multivariable logistic regression analysis was carried out using variables with *p* < 0.05 in addition to age and sex (Table 4). Two models were constructed; only demographic factors were included in Model I and both demographic factors and genetic factors in Model II. In Model 1, overdose, rivaroxaban and anemia were significantly associated with bleeding. In model II, patients with rivaroxaban anemia, *ABCB1* rs3842 variant homozygote (CC) and *APOB* rs13306198 variant allele (AG, AA) had a higher risk of bleeding risk. The Hosmer–Lemeshow test for bleeding revealed a good fit for the final model in both model I and model II (χ^2^ = 0.78 and *p* = 0.94; χ^2^ = 0.63 and *p* = 0.73, respectively).

**Figure 2:**
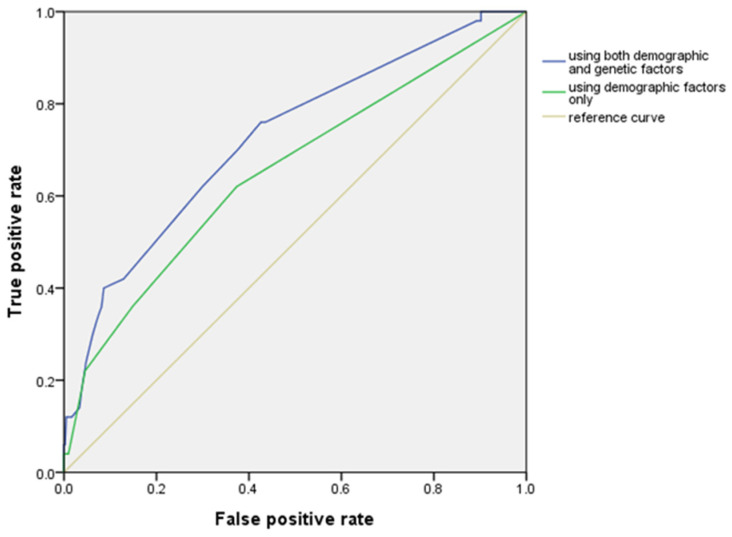
The receiver operating characteristic (ROC) curve for bleeding using demographic factors only and using both demographic factors and genetic factors.

**Figure 3:**
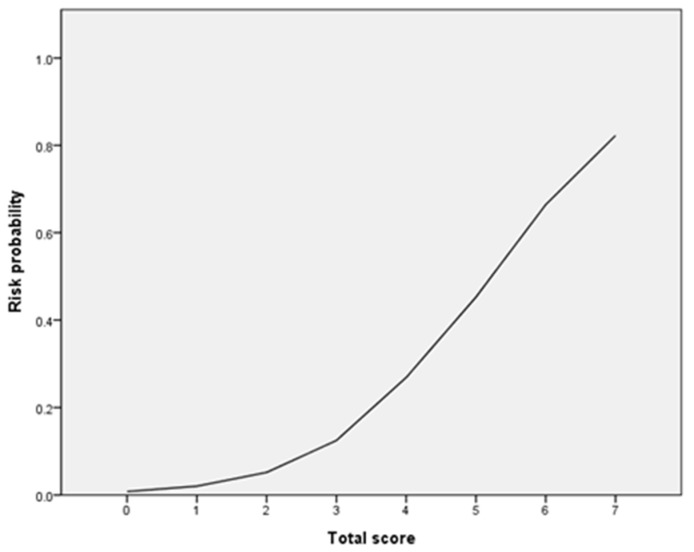
The logistic regression curve of the probability of bleeding versus the proposed scoring scale.

Whereas the area under the receiver operating characteristic curve (AUROC) value using demographic factors only was 0.65 (95% confidence interval (CI): 0.56–0.74), the AUROC increased to 0.72 by adding genetic factors (95% CI: 0.65–0.80) (Figure 2). Therefore, we constructed a risk scoring system using both demographic factors and genetic factors, which remained in Model II; overdose, rivaroxaban, anemia, *ABCB1* rs3842, *APOB* rs693 and *APOB* rs13306198 were taken as 2, 1, 1, 1, 3 and 1 point, respectively. The total risk score ranged from 0 to 8, and there was no patient with a score of 9. The logistic regression curve obtained by mapping the scores to risk scores is shown in Figure 3, and the observed and predicted bleeding risks are presented in Table 5. The observed bleeding risks of patients receiving DOACs with 0, 1, 2, 3, 4, 5, 6, 7 and 8 points were 0.0%, 5.9%, 0.0%, 5.4%, 11.3%, 32.6%, 33.3%, 66.7% and 100.0%, respectively. The predicted bleeding risks of patients receiving DOACs with 0, 1, 2, 3, 4, 5, 6, 7 and 8 points were 0.8%, 2.0%, 5.4%, 4.2%, 12.5%, 26.9%, 47.0%, 64.3% and 82.3%, respectively.

## 4. Discussion

This study revealed that the rs3842 SNP of *ABCB1* was associated with bleeding specifically in patients taking DOACs. The allele substitution of *ABCB1* rs3842, which is a 3ʹ-UTR SNP, might affect protein expression because it disrupts or creates miRNA binding sites [24]. Carriers of the *ABCB1* rs3842 variant allele (C) were found to have lower disease activity scores in a study including patients with rheumatoid arthritis who received methotrexate [25]. Variant allele carriers for *ABCB1* rs3842 exhibited 26% higher efavirenz bioavailability than homozygote of wild-type allele [26]. The mechanism by which rs3842 regulates ABCB1 expression and the resulting effect on treatment response in different population warrant further investigation.

Meanwhile, in a meta-analysis, the C_max_ of DOACs and AUC_0-∞_ of DOACs was lower among *ABCB1* rs1045642 CC carriers and rs2032582 GG carriers compared to those with the TT and A/T allele [27]. In addition, the lower C_max_ was observed among carriers of *ABCB1* compared with those with the A/T allele. In a retrospective real-world study, *ABCB1* rs1045642 was associated with a reduced risk of thromboembolic outcomes [28]. However, in our study, *ABCB1* rs1128503, rs2032582 and rs1045642 were not significantly associated with bleeding, whereas rs3842 of ABCB1 was associated.

Apolipoprotein B is a key structural component of all atherogenic lipoproteins (LDL, VLDL and IDL) [29]. The *APOB* rs13306198 is located in the N-terminal αβ_1_ domain, which forms a lipid pocket necessary for VLDL and chylomicron particle assembly [30]. The effect of *APOB* rs13306198 on anticoagulation activity is controversial, depending on the drugs used or outcomes evaluated. *APOB* rs13306198 was significantly associated with an under-anticoagulation state during the first week (*p* = 0.011) in a study including 252 cardiac valve replacement patients treated with warfarin [31]. The findings are different from our results, possibly due to the use of different drugs.

Although *APOB* rs693 is a synonymous variant, it is related to the circulating concentration of LDL cholesterol. In a meta-analysis of 61 studies including 50,018 subjects, variant allele carriers had high levels of APOB, TG, TC and LDL-C and low HDL-C levels [32]. A meta-analysis including 14 case-control studies showed that *APOB* rs693 may be a risk factor for gallstone disease, especially in Asians [33]. The SNP rs693 in the *APOB* gene increased the risk of breast cancer and aortic stenosis among Chinese subjects [34,35]. Moreover, *APOB* rs693 was associated with the presence of plaque on carotid arteries [36]. In this study, *APOB* rs693 was associated with a 0.15-fold higher risk of bleeding, although statistical significance was not found in the multivariable analysis.

Similar to our research, a study showed that an increased risk of major bleeding was associated with high doses of DOACs (hazard ratio (HR) = 2.19, 95% CI: 1.07–4.46) compared with recommended doses of DOACs [37]. Moreover, the bleeding risk of patients who overdosed on DOACs was more than four times higher (OR = 4.21) in our analysis, which was two times the risk reported in the meta-analysis. Several studies have pointed out that there are some differences in oral anticoagulation treatment between Asian and non-Asian patients with AF for the following reasons: (1) The baseline risks of thromboembolism and bleeding are higher in Asians than in non-Asians; (2) Asians are more vulnerable to anticoagulation related bleeding risk [38]; and (3) Asians generally have greater use of antiplatelets [39]. Moreover, the trough edoxaban concentrations and anti-FXa activities have been found to be lower for Asians compared with non-Asians, even after accounting for the protocol mandated dose reduction [40]. Further studies are required to investigate the association between dose of DOACs and bleeding risk, especially in Asians.

In our study, rivaroxaban was the only and the least safe DOAC, which had almost three times greater bleeding risk than other DOACs. Dabigatran was the safest drug (OR = 0.35) among any anticoagulant in terms of the risk of intracranial hemorrhage (ICH) in a network meta-analysis of 17 randomized controlled trials (RCTs), whereas rivaroxaban was the least safe (OR = 0.44) [41]. Several meta-analyses also showed that rivaroxaban was associated with the highest bleeding risk. Among patients taking rivaroxaban, the risk ratio of postoperative bleeding was higher compared to with that of healthy patients, and the risk of major bleeding and CRNMB was higher compared with that of patients taking apixaban [42,43]. In another meta-analysis, compared with warfarin, apixaban and dabigatran resulted in statistically significant risk reductions in major bleeding, whereas rivaroxaban did not (HR = 0.60, 95% CI: 0.52–0.69; HR = 0.79, 95% CI: 0.70–0.90; HR = 1.03, 95% CI: 0.86–1.22, respectively) [44]. In consideration of the finding of previous studies and our study, more attention is needed when prescribing rivaroxaban.

In our study, 27.6% of the total population (129 patients) had anemia. Anemia has been identified as a strong predictor of bleeding in patients with AF taking anticoagulants [45]. In a meta-analysis of 28 studies encompassing 365,484 AF patients, anemia was associated with a 78% increase in major bleeding and a 77% increase in gastrointestinal bleeding [46]. Additionally, HEMORR_2_HAGEs, ATRIA, RIETE, and CHEST scores include anemia [18,47,48]. Therefore, patients with anemia need to be aware of the potential risks when they consider taking DOACs or during DOAC therapy.

Although there are several bleeding risk scoring systems for anticoagulants, most of them have been primarily validated or evaluated using VKAs and parenteral anticoagulants. No score had good diagnostic accuracy during a validation. For example, a study showed that AUROC of the HAS-BLED was 0.62 [49]. In addition, although the scoring system such as HEMORR2HAGES includes genetic factors such as *CYP2C9* SNP [18], it can be only applied to warfarin, but not DOAC, due to their different metabolic pathways [47]. However, we constructed the model including genetic factors, which had better performance than the model including demographic factors only (AUROC: 0.73 vs. 0.65).

The limitations of this study are related to the retrospective design with the relatively small sample size. Our study included only Asians. In addition, a relatively large number of patients were excluded according to the exclusion criteria. However, there was no significant difference in the characteristics between the included patients and excluded patients. Further prospective large cohort studies are required to validate our findings.

## 5. Conclusions

In conclusion, our study constructed bleeding risk scoring systems using both demographic factors and genetic factors that affected bleeding complications during DOAC therapy. After validation of the results, the constructed models can be used to predict bleeding risks in patients taking DOAC, identify the high-risk patients in advance, and provide dose adjustments or close monitoring. By applying these results in clinical practice, it would be expected to provide patients with more effective and safer anticoagulation therapy in terms of personalized medicine.

## Figures and Tables

**Table 1:** Baseline characteristics of patients included in this study.

Characteristic	Bleeding (n = 50)	No Bleeding (n = 418)	*p*
Sex			0.93
Female	19 (38.0)	156 (37.3)	
Male	31 (62.0)	262 (62.7)	
Age (years)	69.32 ± 9.28	69.12 ± 0.50	0.90
<65	16 (32.0)	125 (29.9)	0.76
≥65	34 (68.0)	293 (70.1)	
BMI (kg/m^2^)	25.48 ± 3.94	24.97 ± 3.38	0.34
<25	22 (45.8)	204 (51.1)	0.49
≥25	26 (54.2)	195 (48.9)	
Creatinine clearance (mL/min)	68.04 ± 25.36	69.08 ± 24.70	0.78
<30	5 (10.4)	18 (4.5)	0.09
≥30	43 (89.6)	384 (95.5)	
AST (IU/L)			0.92
<40	42 (87.5)	344 (88.0)	
≥40	6 (12.5)	7 (12.0)	
ALT (IU/L)			0.39
<40	43 (89.6)	322 (84.9)	
≥40	5 (7.8)	59 (15.1)	
Types of DOACs			0.04
Apixaban	13 (26.0)	168 (40.2)	
Edoxaban	18 (36.0)	141 (33.7)	
Rivaroxaban	16 (32.0)	58 (13.9)	
Dabigatran	3 (6.0)	51 (12.2)	
Prescription dose ^a^			0.01
Underdose	18 (36.0)	133 (31.8)	
Standard dose	28 (56.0)	278 (66.5)	
Overdose	4 (8.0)	7 (1.7)	
Co-medications			
Antiplatelets	3 (6.0)	51 (12.2)	0.20
ACEI or ARBs	19 (38.0)	186 (44.5)	0.38
Beta-blockers	38 (76.0)	295 (70.6)	0.42
Calcium channel blockers	13 (26.0)	116 (27.8)	0.79
Diuretics	11 (22.0)	109 (26.1)	0.53
Statins	28 (56.0)	245 (58.6)	0.72
CYP inducers	0 (0.0)	1 (0.2)	1.00
CYP inhibitors	7 (14.0)	58 (13.9)	0.99
Previous myocardial infarction	4 (8.0)	36 (8.6)	1.00
Previous stroke/TIA/thromboembolism	28 (56.0)	179 (42.8)	0.08
Previous bleeding events	4 (8.0)	18 (4.3)	0.28
Comorbidities			
Atrial fibrillation	49 (98.0)	398 (98.5)	0.78
Hypertension	34 (68.0)	282 (67.5)	0.94
Diabetes mellitus	14 (28.0)	119 (28.5)	0.95
Heart failure	5 (10.0)	78 (18.7)	0.13
Anemia	22 (44.0)	107 (25.6)	0.01
Smoking	6 (12.0)	57 (13.6)	0.75
Alcohol	17 (34.0)	134 (32.1)	0.78
CHA_2_DS_2_-VASc risk of stroke	3.62 ± 1.69	3.47 ± 1.76	0.57
Modified HAS-BLED	2.00 ± 0.95	1.94 ± 1.04	0.70

ACEIs: angiotensin converting enzyme inhibitors; ALT: alanine transferase; AST: aspartate transaminase; ARBs: angiotensin II receptor blockers; BMI: body mass index; CYP: cytochrome P450 family; DOACs: direct oral anticoagulants; TIA: transient ischemic attack. ^a^ Standard dose was defined according to the FDA-approved labeling.

**Table 2:** Effects of *ABCB1* and *CYP3A5* grouped genotypes on bleeding complication.

dbSNP rsID	Grouped Genotype	Bleeding (n = 50)	No Bleeding (n = 418)	*p*
***ABCB1***				
rs3842 (T>C)	TT, CT	40 (80.0)	379 (90.7)	0.02
	CC	10 (20.0)	39 (9.3)	
rs1045642 (A>G)	AA	7 (14.0)	42 (10.1)	0.39
	AG, GG	43 (86.0)	375 (89.9)	
rs2032582 (A>C)	AA, AC	37 (79.0)	331 (79.6)	0.36
	CC	13 (26.0)	85 (20.4)	
rs1128503 (A>G)	AA, AG	40 (80.0)	339 (81.1)	0.85
	GG	10 (20.0)	79 (18.9)	
rs3213619 (A>G)	AA, AG	50 (100.0)	413 (99.5)	1.00
	GG	0 (0.0)	2 (0.5)	
rs3747802 (A>G)	AA	43 (86.0)	368 (88.2)	0.64
	AG, GG	7 (14.0)	49 (11.8)	
***CYP3A5***				
rs776746 (C>T)	CC, CT	46 (92.0)	396 (95.7)	0.28
	TT	4 (8.0)	18 (4.3)	

**Table 3:** Effects of *APOB* and *APOE* grouped genotypes on bleeding complication.

dbSNP rsID	Grouped Genotype	Bleeding (n = 50)	No Bleeding (n = 418)	*p*
***APOB***				
rs1042034 (C>T)	CC	34 (68.0)	233 (55.9)	0.10
	CT, TT	16 (32.0)	184 (44.1)	
rs2163204 (T>G)	TT, GT	50 (100.0)	413 (99.0)	0.49
	GG	0 (0.0)	4 (1.0)	
rs693 (G>A)	GG	49 (98.0)	369 (88.3)	0.04
	AG, AA	1 (2.0)	49 (11.7)	
rs679899 (G>A)	GG, AG	12 (24.0)	119 (28.5)	0.50
	AA	38 (76.0)	298 (71.5)	
rs13306194 (G>A)	GG, AG	49 (98.0)	413 (98.8)	0.49
	AA	1 (2.0)	5 (1.2)	
rs13306198 (G>A)	GG	37 (74.0)	381 (91.1)	<0.01
	AG, AA	13 (26.0)	37 (8.9)	
rs1367117 (G>A)	GG	42 (84.0)	324 (77.5)	0.29
	AG, AA	8 (16.0)	94 (22.5)	
***APOE***				
rs429358 (T>C)	TT	43 (86.0)	329 (79.5)	0.27
	CT, CC	7 (14.0)	85 (20.5)	
rs7412 (C>T)	CC	42 (89.4)	345 (85.2)	0.44
	CT, TT	5 (10.6)	60 (14.8)	

**Table 4:** Univariate and multivariable regression analyses to identify predictors for bleeding.

Predictors	Unadjusted OR (95% CIs)	Model I	Model II
		Adjusted OR (95% CI)	Adjusted OR (95% CI)
Female	1.03 (0.56–1.88)		
Age (≥65)	0.91 (0.48–1.70)		
Overdose	5.11 (1.44–18.10)	4.02 (1.04–15.46) *	4.21 (1.00–17.73)
Rivaroxaban	2.92 (1.52–5.63)	2.70 (1.37–5.33) *	2.59 (1.26–5.30) *
Anemia	2.28 (1.25–4.16)	2.33 (1.26–4.31) **	2.61 (1.37–4.96) **
*ABCB1* rs3842 CC	2.43 (1.13–5.23)		2.44 (1.07–5.58) *
*APOB* rs693 GG	6.49 (0.88–47.62)		6.85 (0.90–52.63)
*APOB* rs13306198 AG, AA	3.62 (1.77–7.41)		3.00 (1.39–6.47) *

CI: confidence interval; OR: odds ratio. Model I included variables of sex, age, overdose, rivaroxaban and anemia. Model II included variables of sex, age, overdose, rivaroxaban, anemia, *ABCB1* rs3842, *APOB* rs693 and *APOB* rs13306198. * *p* < 0.05; ** *p* < 0.01.

**Table 5:** The observed and predicted bleeding risk (%) by risk score.

Score	Bleeding	Total	Observed Bleeding Risk (%)	Predicted Bleeding Risk (%)
0	0	25	0.00	0.79
1	1	17	5.88	2.02
2	0	7	0.00	5.41
3	11	203	5.42	5.18
4	18	160	11.25	12.51
5	14	43	32.56	26.90
6	3	9	33.33	47.00
7	2	3	66.67	64.27
8	1	1	100.00	82.25

## Data Availability

The data presented in this study are available on request from the corresponding author.
